# Detection of a parvovirus and a beak and feather disease virus genome sequence from rose-ringed parakeet (*Psittacula krameri*)

**DOI:** 10.1128/mra.00329-24

**Published:** 2024-07-31

**Authors:** Subir Sarker, Saranika Talukder

**Affiliations:** 1 Biomedical Sciences & Molecular Biology, College of Public Health, Medical and Veterinary Sciences, James Cook University, Townsville, Queensland, Australia; 2 Australian Institute of Tropical Health and Medicine, James Cook University, Townsville, Queensland, Australia; 3 College of Public Health, Medical and Veterinary Sciences, James Cook University, Townsville, Queensland, Australia; Katholieke Universiteit Leuven, Leuven, Belgium

**Keywords:** parvovirus, circovirus, birds

## Abstract

This study reports a genome of psittaciform chaphamaparvovirus 4 (PsChPV-4) and a beak and feather disease virus (BFDV) detected in fecal materials of rose-ringed parakeet. The genomes of PsChPV-4 and BFDV were 4,304 and 2,009 bp long, respectively, and both genomes possessed a genomic structure consistent with their respective genera.

## ANNOUNCEMENT

Chaphamaparvoviruses (ChPVs) are nonenveloped, icosahedral viruses from the *Parvoviridae* family, with a linear single-stranded DNA genome of ~4.0 to 4.5 kb (1). They contain two major genes: a nonstructural (NS) replicase gene and a capsid (VP) gene (2, 3). ChPVs are widespread, detected in the feces of birds (4–7) and mammals (8) and cause renal disease in laboratory mice (9). Recently, ChPVs were detected in the liver of rainbow lorikeets (10) and chickens (11, 12) in Australia.

A beak and feather disease virus (BFDV) from the *Circoviridae* family has a circular single-stranded ~2.0 kb DNA genome (13) and is a dominant viral pathogen of psittacine birds (14, 15), infecting many distantly related Australian avian species (16–19). Here, we report a PsChPV-4 and a BFDV genome sequence from a rose-ringed parakeet.

During routine animal husbandry practice, fecal materials were collected from a group of healthy captive rose-ringed parakeets, housed in a pet shop in Victoria, Australia (37°1′12.36″S, 144°57′52.56″E). The Animal Ethics Committee at La Trobe University was informed that findings from the diagnostic material were to be used in a publication, and a formal waiver of ethics approval was granted. All sample processing was performed following manufacturer’s protocols unless otherwise noted. Viral nucleic acids were extracted using a QIAamp viral RNA minikit (Qiagen, USA). The library was prepared using an Illumina DNA preparation kit, starting with 250 ng of DNA (6). The quality and quantity of the prepared library were assessed using D1000 TapeStation assay and qPCR quantification by the Australian Genome Research Facility (Melbourne, Australia), and the library was sequenced with the Illumina NovaSeq SP sequencing platform, generating 150 bp paired-end reads.

Sequencing data were analyzed as per the established pipeline (20–23) using Geneious Prime (version 2023.1.1, Biomatters, New Zealand). Briefly, 27.26 million raw reads (average read length of 150 nt) were pre-processed to remove the Illumina adapter, ambiguous base calls, and poor-quality reads (trim using quality score, limit 0.05; trim ambiguous nucleotide up to 15), followed by mapping against chicken genome (*Gallus gallus*, GenBank accession no. NC_006088) to remove host DNA. A total of 27.23 million trimmed and unmapped reads were used as input data for *de novo* assembly in Geneious Prime (version 2023.1.1). The resulting contigs were compared against the nonredundant nucleotide database using BLASTN, which identified genomes of PsChPV-4 (4,304 bp, average coverage of 32.88×) and BFDV (2,009 bp of average coverage of 20.03×). Annotation of the assembled genomes was performed using default parameter under the genetic code of standard (transl_table 1) in Geneious Prime (version 2023.1.1). All software was used with default parameters except where stated.

**TABLE 1:** Summary of the detected viruses

Virus name	GenBank accession	Genome length/completeness	G+C content (%)	% of Top BLAST hit (GenBank accession)		% of Top BLAST hit (GenBank accession)	
				NS1	VP1	Replication-associated (Rep) gene	Capsid gene(Cap)
PsChPV-4	OR729118	4,304 nt, no (however, all the coding genes are complete)	66.7	57.74% (QTE03753.1)	53.96% (YP_010802669.1)	NA^*a*^	NA
BFDV	OR729121	2,009 nt, yes	54.1	NA	NA	98.62% (KM887918.1)	98.79% (KY189056.1)

^*a*^ NA, particular gene does not belong to the specific virus.

The PsChPV-4 genome contained four open reading frames (ORFs), and a comparative analysis of the predicted ORFs was conducted by using BLASTX and BLASTP (24) (Table 1). Like other parvoviruses (25, 26), the complete NS1 gene of PsChPV-4 was 675 amino acids in length and encodes the helicase, including the conserved ATP- or GTP-binding Walker A loop (GPxNTGKT/S; _314_**GP**S**NTGKS**_321_), Walker B (xxxWEE; _353_IGI**WEE**_358_) Walker B’ (KQxxEGxxxxxPxK; _370_**KQ**VL**EG**MTTSI**P**V**K**_383_), and Walker C (PxxxTxN; _394_**P**IFI**T**T**N**_400_) aa motifs. In addition, the NS1 protein contains two conserved replication initiator (endonuclease) motifs, xxHuHxxxx (VF_104_**H**V**H**_110_VLLR) and YxxK (_166_**Y**LI**K**_169_) (conserved amino acids are indicated in bold letters, and “u” indicates a hydrophobic residue).

The BFDV genome contained two ORFs, and comparative analysis of the predicted ORFs was conducted by using BLASTX and BLASTP (24) (Table 1).

This study highlights the evidence of a parvovirus and a BFDV in a healthy captive rose-ringed parakeet. This expands the host range of PsChPV and suggests that at least some ChPVs may have a wide host range.

## Data Availability

The complete viral genome sequences from this study have been deposited in DDBJ/ENA/GenBank under the accession nunmbers OR729118.1 and OR729121.1. The raw sequencing data from this study have been deposited in the NCBI Sequence Read Achieve (SRA) under the accession number SRR26413812 (BioProject accession number PRJNA1028305).
